# Synaptic mitochondria in aging and neurodegenerative diseases: Functional decline and vulnerability

**DOI:** 10.4103/NRR.NRR-D-24-01571

**Published:** 2025-06-19

**Authors:** Karina A. Cicali, Angie K. Torres, Cheril Tapia-Rojas

**Affiliations:** 1 Laboratory of Neurobiology of Aging, Centro Científico y Tecnológico de Excelencia Ciencia & Vida, Fundación Ciencia & Vida, Santiago, Chile; 2 Facultad de Medicina y Ciencia, Universidad San Sebastián, Santiago, Chile; 3 Centro de Excelencia en Biomedicina de Magallanes (CEBIMA), Universidad de Magallanes, Punta Arenas, Chile

**Keywords:** aging, hippocampus, memory, mitochondria, synaptic mitochondria

## Abstract

Aging is a physiological and complex process produced by accumulative age-dependent cellular damage, which significantly impacts brain regions like the hippocampus, an essential region involved in memory and learning. A crucial factor contributing to this decline is the dysfunction of mitochondria, particularly those located at synapses. Synaptic mitochondria are specialized organelles that produce the energy required for synaptic transmission but are also important for calcium homeostasis at these sites. In contrast, non-synaptic mitochondria primarily involve cellular metabolism and long-term energy supply. Both pools of mitochondria differ in their form, proteome, functionality, and cellular role. The proper functioning of synaptic mitochondria depends on processes such as mitochondrial dynamics, transport, and quality control. However, synaptic mitochondria are particularly vulnerable to age-associated damage, characterized by oxidative stress, impaired energy production, and calcium dysregulation. These changes compromise synaptic transmission, reducing synaptic activity and cognitive decline during aging. In the context of neurodegenerative diseases such as Alzheimer’s, Parkinson’s, and Huntington’s, the decline of synaptic mitochondrial function is even more pronounced. These diseases are marked by pathological protein accumulation, disrupted mitochondrial dynamics, and heightened oxidative stress, accelerating synaptic dysfunction and neuronal loss. Due to their specialized role and location, synaptic mitochondria are among the first organelles to exhibit dysfunction, underscoring their critical role in disease progression. This review delves into the main differences at structural and functional levels between synaptic and non-synaptic mitochondria, emphasizing the vulnerability of synaptic mitochondria to the aging process and neurodegeneration. These approaches highlight the potential of targeting synaptic mitochondria to mitigate age-associated cognitive impairment and synaptic degeneration. This review emphasizes the distinct vulnerabilities of hippocampal synaptic mitochondria, highlighting their essential role in sustaining brain function throughout life and their promise as therapeutic targets for safeguarding the cognitive capacities of people of advanced age.

## Introduction

Aging is a process that involves accumulative deterioration of cellular and physiological functions, including those associated with the brain (Guo et al., 2022; Lee and Kim, 2022; Aguilar-Hernandez et al., 2023; Lopez-Otin et al., 2023). This deterioration significantly impacts the hippocampus, a region critical for spatial and recognition learning and memory (Zhang et al., 2021; Hayes et al., 2024). Over time, the hippocampus experiences a decline in its capacities due to cellular alterations, synaptic loss, and neuronal apoptosis (Tucsek et al., 2017; Nadal et al., 2020; Liu et al., 2023; Zhou et al., 2024). Unlike other eukaryotic cells, neurons possess a unique structure with extensive prolongations extending from the cell body and establishing connections through synapses (Sudhof, 2021). These synapses are critical regions for information exchange, where effective transmission depends on a continuous supply of energy and precise control of calcium (Ca²⁺) levels mainly provided by the mitochondria (Gleichmann and Mattson, 2011). However, disruption in these processes impedes neuronal communication, affecting synaptic function and, consequently, the proper functioning of the hippocampus, resulting in age-related memory decline (Cicali and Tapia-Rojas, 2024).

Synaptic mitochondria are essential organelles located at synapses, specific communication sites between neurons where signal transmission occurs. This mitochondrial population is different from mitochondria located in the soma and neurites because these mitochondria are adapted to meet high energy demands and regulate Ca²⁺ levels to sustain neurotransmission and synaptic plasticity (Datta and Jaiswal, 2021; Verma et al., 2022; Duarte et al., 2023; Samanta et al., 2025). They participate in and regulate critical processes such as neurotransmitter exocytosis, vesicular reuptake, and Ca²⁺ buffering during neuronal activity, which ensures the stability and efficiency of communication between neurons (Ly and Verstreken, 2006; Mattson et al., 2008; Myeong et al., 2024). Moreover, these mitochondria play a crucial role in regulating reactive oxygen species (ROS) levels to protect synapses from oxidative stress. Also, mitochondrial ROS modulates synaptic activity by recruiting glutamate receptors to synaptic compartments (Doser et al., 2024) and controlling the synaptic pruning according to synaptic activity (Cobley, 2018).

In contrast to the high specificity, restringed localization, and functional demand, non-synaptic mitochondria are mainly found in the soma, the perinuclear region, and non-synaptic areas of neuronal extensions and have more general functions. These mitochondria are primarily responsible for cellular metabolism and basal energy maintenance (Perkins et al., 2001; Hill et al., 2018; Seager et al., 2020). Thus, the differences in location and function highlight the specialization of synaptic mitochondria to sustain neuronal communication and preserve synaptic function; however, in aging and neurodegenerative diseases, synaptic mitochondria fail previous to non-synaptic mitochondria, are more vulnerable to nocive stimulus, and their dysfunction correlates with the age-related memory impairment (Olesen et al., 2020).

In neurons, mitochondrial biogenesis leading to the production of new mitochondria occurs primarily in the soma by a mechanism mediated by the PGC-1α/NRF/TFAM pathway. Then, these organelles are dynamically transported across neuronal microtubules by a highly regulated system (Cicali and Tapia-Rojas, 2024). Anterograde transport moves mitochondria from the soma to distal regions, including the synapse, whereas retrograde transport returns damaged mitochondria to the soma for reparation, replacement, and/or degradation (Saxton and Hollenbeck, 2012; Cicali and Tapia-Rojas, 2024). This balance between the two fluxes is essential to maintain a functional mitochondrial population at synapses and to meet local metabolic needs (Badal et al., 2019; Duarte et al., 2023). Mitochondrial trafficking is mediated by a specialized protein complex involving trafficking kinesin 1/2 (TRAK1/2), Milton, and mitochondrial Rho GTPase (Miro-1). TRAK1/2 proteins act as adaptors, connecting the molecular motors kinesin and dynein/dynactin to Miro, allowing the mitochondrial transport bi-directionally along microtubules (Schwarz, 2013; Duarte et al., 2023). This complex transport system allows mitochondria to be strategically positioned at synapses, contributing to neuronal transmission by producing adenosine triphosphate (ATP) and regulating Ca²⁺ levels (Cicali and Tapia-Rojas, 2024). Their proper localization is crucial for correct synaptic functioning, as any alteration in mitochondrial trafficking or positioning can lead to synaptic dysfunctions, neurodegeneration, and age-related diseases (Brickley and Stephenson, 2011; Calkins et al., 2011; van Spronsen et al., 2013; Lopez-Domenech et al., 2016; Loss and Stephenson, 2017).

Nevertheless, this back-and-forth movement of newly synthesized mitochondria toward the synapses and the return of damaged mitochondria to the soma is energetically costly in neurons with longer projections like motor neurons (Cardanho-Ramos and Morais, 2021). For this reason, it is also suggested that mitochondrial biogenesis must occur in distal regions, including synapses, by an alternative mechanism that involves Ca^2+^ signaling since PGC-1α and NRF1/2 function exclusively in the nucleus (Atkins et al., 2004). Thus, local mitochondrial biogenesis may ensure a continuous supply of functional mitochondria in synaptic regions with high energy demand (Muller et al., 2005; Li et al., 2020; Cardanho-Ramos et al., 2024); however, is possible that these mechanisms will be damaged in aging, contributing to the more severe mitochondrial dysfunction at synapses at an advanced age or at the synaptic mitochondrial depletion observed in neurodegenerative diseases such as AD.

This review focuses on the importance of synaptic mitochondria, analyzing the main differences between synaptic mitochondria and non-synaptic mitochondria in terms of their structural and functional characteristics. It addresses how synaptic mitochondria are specifically adapted to respond to the high energy requirements and precise regulation of calcium at synapses, as opposed to non-synaptic mitochondria that play more general roles in cellular metabolism. Also, how aging and neurodegenerative diseases influence the synaptic mitochondrial function will be explored, highlighting how dysfunction of these mitochondria contributes to cognitive decline and increases neuronal vulnerability. Additionally, we discuss the particular vulnerability of synaptic mitochondria, which, due to their specialized location and functions, are especially susceptible to alterations that may compromise synaptic communication and contribute to cognitive impairment in aging and age-related pathologies.

## Search Strategy

We conducted a comprehensive search in the PubMed database, covering articles published from 2000–2024, using the following Medical Subject Headings (MeSH) terms: (“mitochondria” [Mesh] AND (“synapses” [Mesh] OR “synaptic mitochondria” [Mesh] OR “mitochondria” [Mesh])) AND (“hippocampus” [Mesh] OR “synaptic mitochondria” [Mesh]) AND (“memory”[Mesh] OR “hippocampal synaptic mitochondria” [Mesh]). Title and abstract screening focused on studies exploring the characteristics of mitochondria at synapses, the relevance of synaptic mitochondria in the hippocampus, and its role in the memory processes. While our search covered a broad time frame, we prioritized recent literature published between 2019 and 2024. Key earlier studies and reviews of significant relevance were also included.

## Synaptic Mitochondria Are Crucial to Maintaining Synaptic Architecture and Communication

**Figure 1:**
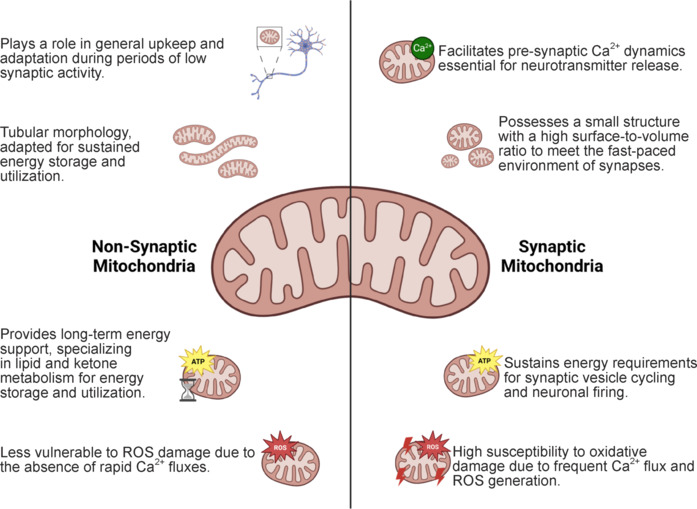
Comparison of the functions of non-synaptic and synaptic mitochondria. Schematic representation, which highlights key differences in mitochondrial function and morphology. Non-synaptic mitochondria (left side) possess a more elongated and interconnected morphology. They are mainly involved in the overall regulation of cellular metabolism, mitochondrial dynamics, and global energy support. In contrast, synaptic mitochondria (right side) present a generally more fragmented and compact morphology, are closely related to Ca^2+^ homeostasis, localized ATP production for the energetic demands of synaptic transmission, and are more vulnerable to oxidative damage caused by ROS generation. Created with BioRender.com. ATP: Adenosine triphosphate; ROS: reactive oxygen species.

Synaptic and non-synaptic mitochondria embody highly specialized adaptations that meet the diverse metabolic and functional demands within neurons, accounting for the complex distribution and regulation of energy in different neuronal compartments (Morton et al., 2021; **Figure 1**). Synaptic mitochondria have a structure capable of adapting to the immediate demands of neurotransmission. These mitochondria tend to be smaller, with a high surface-to-volume ratio that enables rapid Ca^2+^ uptake, a crucial feature for responding to the fast-paced environment of the synapse during neuronal firing (Fedorovich et al., 2017; Graham et al., 2017; Cserep et al., 2018; Hill et al., 2018; Seager et al., 2020). This rapid Ca^2+^ uptake, primarily mediated by the mitochondrial calcium uniporter (MCU) (Gherardi et al., 2024; Li et al., 2025; Murphy and Eisner, 2025), allows synaptic mitochondria to support pre-synaptic Ca^2+^ dynamics, a requirement for sustaining neurotransmitter release (Rizzuto et al., 2012). However, this advantage also makes synaptic mitochondria particularly vulnerable to oxidative damage, as the frequent flux of Ca^2+^ generates ROS, placing synaptic mitochondria at higher risk of dysfunction (Lores-Arnaiz et al., 2016; Olesen et al., 2020). This increased vulnerability to oxidative stress underscores a trade-off between the efficiency of Ca^2+^ handling and the susceptibility to metabolic strain.

In contrast, non-synaptic mitochondria have distinct structural and functional characteristics that reflect a role more attuned to maintaining cellular homeostasis and supporting steady metabolic functions (Volgyi et al., 2015). These mitochondria often exhibit tubular morphology and are enriched in proteins related to the metabolism of lipids and ketone, which suggests an adaptation to longer-term energy storage and utilization rather than immediate energy release (Volgyi et al., 2015; Hill et al., 2018; Seager et al., 2020). This proteomic profile indicates that non-synaptic mitochondria may not face the same rapid Ca^2+^ fluxes as their synaptic counterparts and are, therefore, less susceptible to the damaging effects of ROS, supporting cellular resilience over rapid response (Brown et al., 2006; Naga et al., 2007; Stauch et al., 2014; Lores-Arnaiz et al., 2016; Olesen et al., 2020). This configuration aligns non-synaptic mitochondria with roles that extend beyond the immediate demands of synaptic activity, possibly functioning in general neuronal maintenance and preparation for metabolic shifts, especially during periods of low synaptic activity (Mattson et al., 2008).

Beyond these fundamental distinctions, the localization of mitochondria within specific neuronal subdomains further refines their functional roles (Wong-Riley, 1989). Mitochondria in axons, for example, display higher mobility than those located in dendrites, facilitating swift energy distribution along the long axonal processes and allowing rapid responses to localized energy needs (Fu et al., 2017; Rangaraju et al., 2019; Seager et al., 2020; Mandal et al., 2021; Yang et al., 2023). This mobility is especially relevant for neurons with extensive axonal projections, where energy demands can be widely dispersed. Furthermore, dendritic mitochondria tend to be stationary, supporting the post-synaptic architecture and maintaining the structural integrity of dendritic spines (Fu et al., 2017; Rangaraju et al., 2019; Seager et al., 2020). This strategic localization of mitochondria supports axonal and dendritic functions, demonstrating how mitochondrial positioning is a crucial aspect of neuronal physiology.

Neurons contain a high amount of mitochondria due to their importance to the proper neuronal function (Misgeld and Schwarz, 2017). These organelles play a pivotal role in meeting the high energy requirements of the brain, which consumes approximately 20% of the body’s total energy budget (Rossi and Pekkurnaz, 2019). While glycolysis plays a substantial role in ATP generation during neuronal rest, oxidative phosphorylation becomes the dominant energy production pathway during active synaptic transmission (Fox et al., 1988; Rangaraju et al., 2014). The role of mitochondria in oxidative phosphorylation ensures the sustained ATP production needed for action potentials and synaptic vesicle recycling (Harris et al., 2012). This capacity for dynamic adaptation reflects the role of mitochondria as energy providers and as essential regulators of neuronal activity, responding to energy demands through processes like mitochondrial fission, fusion, and transport (Reddy et al., 2011; Misgeld and Schwarz, 2017; Pekkurnaz and Wang, 2022). Precisely, mitochondrial dynamics through fusion and fission, regulated by protein GTPases, control the morphology of the mitochondrial network (Chen et al., 2023; Grel et al., 2023). A proper balance between these processes is necessary to maintain the connectivity and shape of mitochondria. The mitochondrial network will be more connected if fusion is favored, whereas the mitochondrial architecture will be fragmented if fission is favored. Under normal conditions, these processes are balanced, but one may predominate depending on the cell’s needs (Chen et al., 2023; Grel et al., 2023). Studies in neuronal cultures have shown that manipulating proteins involved in mitochondrial fusion or fission processes can alter the structure and function of synapses and dendritic spines. Specifically, cultured hippocampal neurons with increased expression of Opa1 engaged in the fusion process or reduced expression of Drp1 involved in fission, decreased synaptic density, and dendritic growth (Li et al., 2004). In contrast, the overexpression of Drp1 in primary and organotypic cultures increased the dendritic spine density (Li et al., 2004). Likewise, a decrease in spine formation and maintenance, as well as a decrease in dendritic growth, was observed in mixed cultures from the cerebellum of postnatal mitofusin-2 (Mfn-2) knock-out mice (Chen et al., 2007). This same phenotype was observed in cultures of retinal tissue from Opa1 heterozygous mice (Chen et al., 2007; Williams et al., 2010). Thus, these data strongly indicate that mitochondrial fission is critical to mitochondrial positioning at synapses and, here, promoting dendritic spine density and dendritic growth.

**Figure 2:**
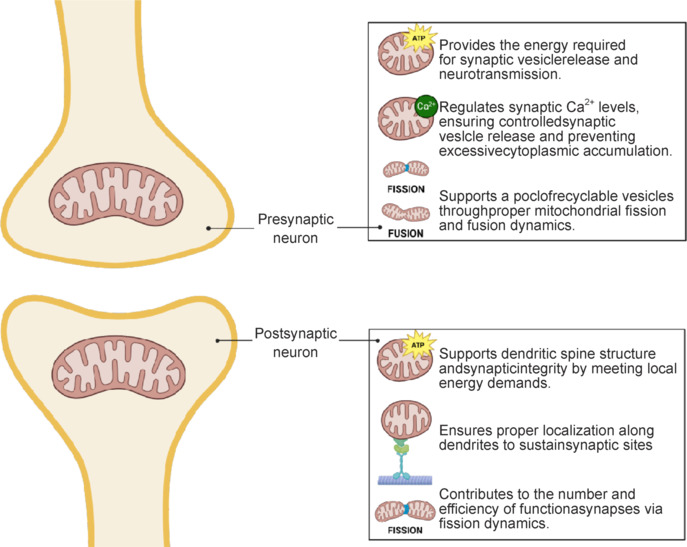
Functions of mitochondria in the pre-and post-synaptic region. Schematic representation of mitochondrial functions in the pre-synaptic and post-synaptic region. In the pre-synaptic region (upper part), mitochondria are essential for producing ATP required for neurotransmitter release processes and regulating Ca^2+^ homeostasis, a key element for synaptic exocytosis of vesicles containing neurotransmitters. Its involvement in mitochondrial fission for adaptation to high local energy demands is also shown. In the post-synaptic zone (lower part), mitochondria are essential for generating ATP that supports synaptic receptor-mediated intracellular signaling processes and maintains synaptic plasticity. Both locations reflect the functional and morphological specialization of mitochondria in synaptic transmission. Created with BioRender.com. ATP: Adenosine triphosphate.

Synaptic mitochondria play distinct roles in the pre-synaptic and post-synaptic regions, reflecting the specialized demands of these compartments (**Figure 2**). In the pre-synaptic terminal, mitochondria serve as powerhouses that sustain neurotransmitter release by providing ATP and regulating Ca^2+^ dynamics (Rangaraju et al., 2019). Mitochondrial dysfunction at this site can severely impair neurotransmission. Inhibition of oxidative phosphorylation, for example, reduces synaptic vesicle release, directly linking mitochondrial ATP production to the efficiency of synaptic transmission (Ivannikov et al., 2013). Effective Ca^2+^ buffering by pre-synaptic mitochondria is critical for controlling calcium levels within the terminal (Billups and Forsythe, 2002). A lack of functional mitochondria at the pre-synaptic zone can lead to excessive cytoplasmic Ca^2+^ accumulation, which increases synaptic vesicle release unpredictably and can result in excitotoxicity (Vaccaro et al., 2017). Morphological changes in pre-synaptic mitochondria, such as transient excessive fusion, can enhance their capacity to buffer Ca^2+^ in periods of synaptic activity, highlighting the functional influence of mitochondrial dynamics on synaptic activity (Kowaltowski et al., 2019). However, studies on mice deficient in the fission protein Drp1 reveal the importance of mitochondrial fission in maintaining a pool of recyclable vesicles, which leads to reduced size and recycling capacity of vesicles—a deficit partially reversible by improving Ca^2+^ buffering capacity (Singh et al., 2018). Thus, these studies reveal the relevance of maintaining a delicate balance between the fission and fusion processes, where mitochondrial fission is necessary to basal mitochondrial function but that, according to synaptic activity, requires transient fusion that later returns to smaller mitochondria in the pre-synaptic compartment to mediate calcium homeostasis and consequently the neurotransmitter release.

In the post-synaptic region, mitochondria, primarily located at dendritic shafts, provide the essential local energetic support for synaptic functions (Oliver and Reddy, 2019; Thomas et al., 2023). They are fundamental for maintaining dendritic spine structure and the integrity of the synapses, with disruptions in mitochondrial function leading to spine and synapse loss (Schuman and Chan, 2004). Proper distribution of mitochondria along dendrites is critical for supporting synaptic sites, with fusion and fission processes ensuring that mitochondria are present to meet local energy demands. In this synaptic compartment, Drp1-dependent mitochondrial fission plays a fundamental role in maintaining an adequate pool of mitochondria in the post-synaptic region that ultimately regulates the number of functional synapses (Li et al., 2004).

The differences, at morphological and functional levels, between synaptic and non-synaptic mitochondria thus reflect a broader adaptation of mitochondria to meet the specific needs of neuronal compartments. Synaptic mitochondria are optimized for rapid response to Ca^2+^ fluctuations and high ATP demand at the synapse. In contrast, non-synaptic mitochondria are positioned to support longer-term cellular maintenance and metabolic stability. This division of mitochondrial labor is essential for understanding neuronal physiology and is sustained by a differential mitochondrial proteome. It provides a framework for examining how mitochondrial dysfunction in specific compartments may contribute to the pathology of neurodegenerative diseases. The intricate balance of mitochondrial dynamics, energy distribution, and localized function illustrates the fundamental role of mitochondria as both powerhouses and regulatory hubs in neuronal health.

## Synaptic Mitochondria in Aging and Neurodegeneration

**Figure 3:**
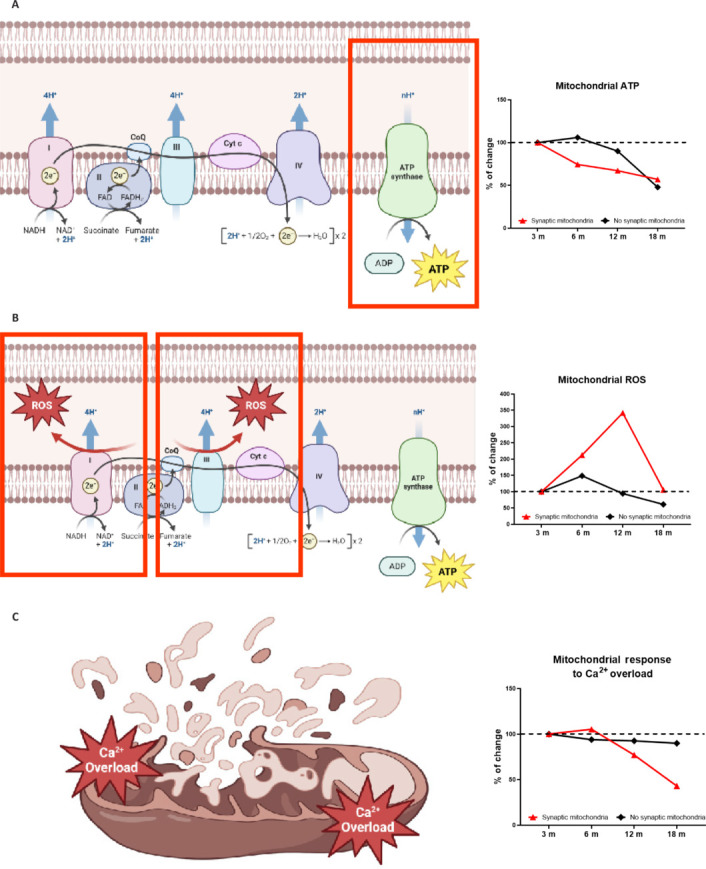
Differential decrease of mitochondrial function in synaptic and non-synaptic mitochondria in aging. Schematic representation and graphs showing the assessment of mitochondrial function in synaptic and non-synaptic mitochondria during aging, measuring three key parameters: (A) mitochondrial ATP production as an indicator of bioenergetic capacity (top panel), (B) mitochondrial ROS production as a marker of oxidative stress (middle panel), and (C) response to Ca^2+^ overload as a measure of swelling predisposition (bottom panel). Data are presented as relative percentage changes at different ages (3, 6, 12, and 18 months), allowing for a comparison of the differential functionality between these two types of mitochondria over time. Created with BioRender.com and GraphPad Prism. ATP: Adenosine triphosphate; m: months; ROS: reactive oxygen species.

**Figure 4:**
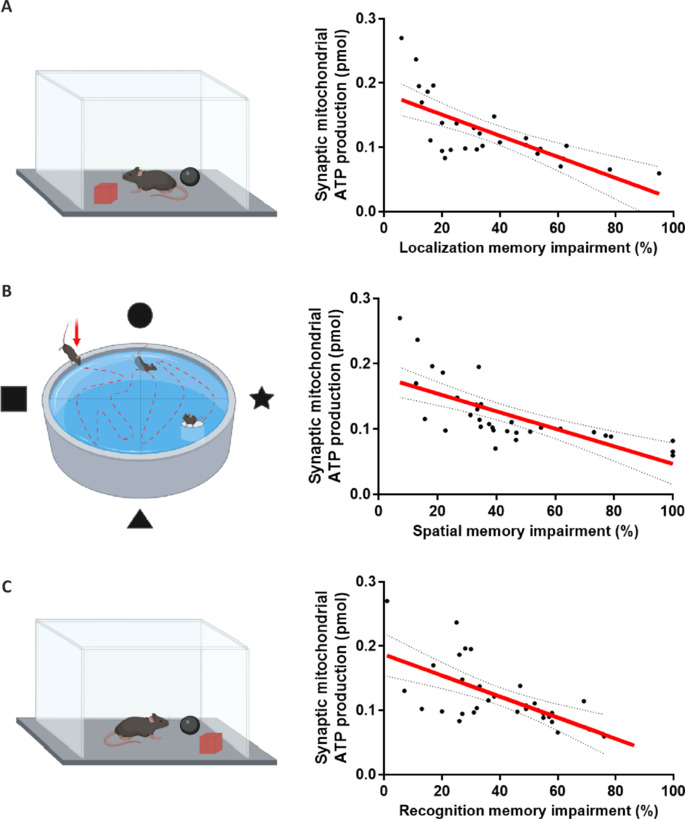
Correlation between decreased function of synaptic mitochondria and hippocampus-dependent cognitive impairment. Schematic representation and graphs that correlate the mitochondrial ATP production (pmol) by synaptic mitochondria with three types of memory: (A) spatial memory, assessed by the Morris water maze test (top panel); (B) recognition memory, assessed by an object recognition test (middle panel); and (C) localization memory, measured by an object location test (bottom panel). Scatter plots show significant negative correlations between ATP production in synaptic mitochondria and levels of impairment in each cognitive domain, indicating that reduced function of synaptic mitochondria is associated with more severe memory impairment. Created with BioRender.com and GraphPad Prism. ATP: Adenosine triphosphate.

The comparison between synaptic mitochondrial dysfunction in aging and neurodegenerative diseases reveals shared mechanisms and critical distinctions in the extent and impact of mitochondrial damage on neuronal function. In healthy aging, synaptic mitochondria exhibit increased vulnerability to damage, possibly due to the accumulation of oxidative stress and age-related insults. As aging progresses, synaptic mitochondria become increasingly dysfunctional, showing early signs of bioenergetic defects, such as reduced ATP production, increased generation of ROS, and heightened sensitivity to calcium overload (**Figure 3**; Olesen et al., 2020; Graham et al., 2021; Samanta et al., 2025). These defects lead to synaptic and memory impairments, particularly in the hippocampus, an area crucial for learning and memory (Todorova and Blokland, 2017; Olesen et al., 2020). Studies in mice previously performed by our group have demonstrated that cognitive decline begins to manifest after 12 months of age, with spatial and recognition memory deteriorating simultaneously with the dysfunction of synaptic mitochondria (**Figure 4**). At 18 months, both synaptic and non-synaptic mitochondria exhibit bioenergetic impairments, but synaptic mitochondria are disproportionately affected, also displaying a greater susceptibility to swelling and damage (Olesen et al., 2020; **Figure 4**).

Interestingly, although synaptic mitochondrial function declines with age, there is no significant depletion of synaptic mitochondria in healthy aging (Torres et al., 2021a). Instead, synaptic mitochondria maintain their distribution across synapses, but their structural integrity deteriorates, with observed alterations such as membrane disruption, reduced electron density, and swelling (Torres et al., 2021a). These structural changes correlate with functional deficits, ultimately contributing to the age-associated cognitive decline in elderly individuals (Olesen et al., 2020). Moreover, synaptic mitochondria derived from the brains of aged animals, such as the hippocampus and cerebral cortex, accumulate higher levels of oxidative damage than non-synaptic mitochondria (Martinez et al., 1996; Brown et al., 2006). This damage increases mitochondrial DNA mutations, reduces bioenergetic function, and increases susceptibility to calcium-induced depolarization (Brown et al., 2006; Stauch et al., 2014; Olesen et al., 2020). While non-synaptic mitochondria resist such dysfunctions and oxidative damage, synaptic mitochondria show declining oxygen consumption rates and increased hydrogen peroxide production, particularly in aged animals, contributing to compromised function (Lores-Arnaiz and Bustamante, 2011; Lores-Arnaiz et al., 2016).

In addition to functional impairments, aging induces structural changes in the mitochondria. Observations of our laboratory in the CA1 region of the hippocampus of aged mice reveal increased mitochondrial area, higher prevalence of swollen mitochondria, and reduced membrane integrity (Torres et al., 2021a). Despite these alterations, synapses with mitochondria in the pre- and post-synaptic region remain unchanged, indicating that synaptic mitochondria are not depleted with age but exhibit severe damage (Torres et al., 2021a). Ultrastructural analysis shows that synaptic mitochondria in the CA1 of aged hippocampus still supply multiple synapses, mirroring the arrangement observed in younger brains. However, these mitochondria often display disrupted membranes and reduced electron density, which agree with compromised function (Cicali and Tapia-Rojas, 2024).

In contrast, in neurodegenerative diseases such as Alzheimer’s disease (AD), Parkinson’s disease (PD), and Huntington’s disease (HD), synaptic mitochondrial dysfunction is more severe and occurs earlier in the disease progression, directly influencing the onset and progression of these disorders. In AD, for example, synaptic mitochondria are significantly impacted by the accumulation of amyloid-beta (Aβ) peptide (Torres et al., 2021b) and phosphorylated tau protein (Torres et al., 2022), known hallmarks of the disease. This accumulation disrupts mitochondrial function by increasing oxidative stress, impairing calcium buffering, and causing morphological damage to mitochondria (Torres et al., 2021a, b; Tracy et al., 2022; Jia et al., 2023; Liang et al., 2024; Daniel Estrella et al., 2025). Proteomic studies have confirmed that mitochondrial dysfunction is one of the earliest detectable abnormalities in AD, with significant alterations in proteins involved in oxidative phosphorylation, the tricarboxylic acid cycle, and mitochondrial electron transport chain complexes (Liang et al., 2024). Aβ and tau accumulation in synaptic mitochondria is particularly harmful because it alters mitochondrial bioenergetics and accelerates synaptic degeneration (Oliver and Reddy, 2019; Jia et al., 2023; Daniel Estrella et al., 2025). Different studies have shown that mitochondrial dysfunction in AD occurs early in the disease, often before other pathological features such as Aβ plaques and tau tangles are detectable. This early mitochondrial dysfunction leads to synaptic failure, including the loss of synaptic proteins, dendritic spines, and synapses (Cai and Tammineni, 2017; Wang et al., 2023). These changes are thought to be a key factor in the cognitive decline and memory deficits observed in AD patients. Moreover, the depletion of mitochondria at synapses, the reduction of synaptic sites, and the disruption of synaptic transmission contribute directly to the cognitive impairments that characterize the disease (Pham et al., 2010; Pickett et al., 2018). As synaptic mitochondria become dysfunctional, they also contribute to mitochondrial fragmentation, a phenomenon observed in AD but not in normal aging, that further exacerbates the decline of synaptic function (Calkins et al., 2011; Du et al., 2017; Morton et al., 2021; Lee et al., 2022; Bhatti et al., 2023).

In PD, the situation is different but similarly marked by mitochondrial dysfunction. Dopaminergic neurons in the substantia nigra, which are critical for motor control, rely heavily on mitochondrial function to maintain their high energy demands (Puopolo et al., 2007). These neurons consume large amounts of ATP to regulate calcium influx, and the generation of oxidative stress is a byproduct of this energy-intensive process (Guzman et al., 2010). In PD, damaged mitochondria accumulate at synaptic terminals, leading to disruptions in mitochondrial dynamics, including impaired mitochondrial transport. These alterations impair the energy supply to synapses, resulting in synaptic dysfunction and neuronal damage (Wang et al., 2011). In PD, the failure of mitophagy, the process by which damaged mitochondria are selectively removed, contributes to the accumulation of dysfunctional mitochondria at synaptic sites, further exacerbating cellular damage (Geisler et al., 2010; Guzman et al., 2010). The dysregulation of mitophagy in PD is linked to mutations in key proteins such as PINK1 and Parkin, key factors for the main mitochondrial degradation route (Kitada et al., 1998; Valente et al., 2004). Without proper mitophagy, dysfunctional mitochondria accumulate at synapses, leading to synaptic failure, neuronal death, and motor deficits.

In HD, mitochondrial dysfunction plays a similarly crucial role in disease progression (Damiano et al., 2010). The mutation of the huntingtin gene leads to the expression of a toxic protein (mutant huntingtin or mHTT) that interferes with mitochondrial dynamics, particularly mitochondrial trafficking. In the early stages of HD, before overt neuronal death and synaptic degeneration, mHTT expression blocks mitochondrial transport to synapses, impairing the energetic support required for synaptic function (Trushina et al., 2004). This disruption of mitochondrial trafficking contributes to ATP depletion at the synapse and increases the risk of excitotoxicity, mainly through abnormal activation of NMDA receptors (Garcia-Larrea et al., 1997; Panov et al., 2002; Fan and Raymond, 2007). The increase of fragmented mitochondria in the neuronal body due to an imbalance in mitochondrial dynamics further contributes to the progression of the disease (Shirendeb et al., 2012; Kim et al., 2014). The resultant synaptic degeneration and excitotoxicity are thought to be central to the cognitive and motor deficits characteristic of HD.

Thus, while aging and neurodegenerative diseases share common features of mitochondrial dysfunction, the severity and timing of the dysfunction differ significantly. In aging, mitochondrial dysfunction in synapses contributes to cognitive decline through progressive structural and functional damage, but synaptic mitochondria are not lost, and the overall decline is more gradual. In contrast, in neurodegenerative diseases, synaptic mitochondrial dysfunction occurs much earlier and more acutely, directly contributing to the pathogenesis of these diseases. The accumulation of pathological proteins and impaired mitochondrial dynamics in diseases such as AD, PD, and HD accelerates synaptic damage, leading to irreversible neuronal loss and significant cognitive and motor impairments. Therefore, preserving and restoring mitochondrial function at synapses may represent a promising therapeutic target for slowing or halting the progression of age-related cognitive decline and neurodegenerative diseases.

## Why Are Synaptic Mitochondria More Vulnerable?

**Figure 5:**
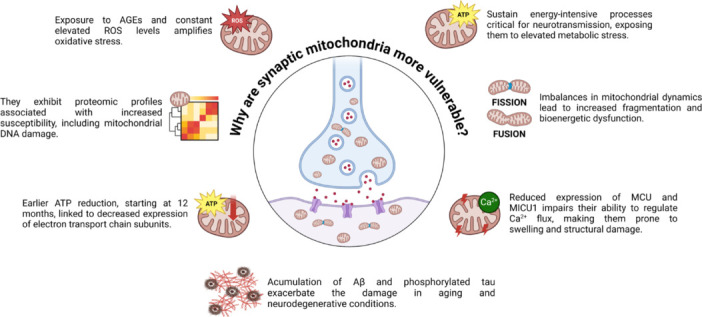
Characteristics that make synaptic mitochondria more vulnerable. Schematic representation of the features that make synaptic mitochondria more susceptible to damage in aging and neurodegeneration. These include their exposure to high levels of ROS and metabolic stress due to high ATP demand, imbalances in mitochondrial dynamics (reduction of fusion proteins such as Mfn1 and Opa1 and increase of fission proteins such as DNM1L), decreased ability to handle Ca^2+^ overloads due to low expression of MCU and MICU1, accumulation of mitochondrial DNA damage, and sensitivity to external insults such as AGEs, as well as accumulation of beta-amyloid and phosphorylated tau inside synaptic mitochondria. Created with BioRender.com. Aβ: Amyloid-beta; AGEs: advanced glycation end products; ATP: adenosine triphosphate; DNM1L: dynamin-1-like protein; MCU: mitochondrial calcium uniporter; Mfn1: mitofusin 1; MICU1: mitochondrial calcium uptake 1; Opa1: optic atrophy protein 1; ROS: reactive oxygen species.

Synaptic mitochondria exhibit heightened vulnerability due to their critical role in supporting the energy-intensive processes required for synaptic function in the brain (Evans et al., 1992; Billups and Forsythe, 2002; Li et al., 2004; Vos et al., 2010; Han et al., 2020; Datta and Jaiswal, 2021; **Figure 5**). This susceptibility arises from their exposure to elevated metabolic demands and intrinsic characteristics that impact their structure, functionality, and regulatory mechanisms. Consequently, synaptic mitochondria are more prone to functional deterioration than their non-synaptic counterparts, as they must contend with internal and external stressors (Quiroz-Baez et al., 2013; Stauch et al., 2014; Olesen et al., 2020).

Proteomic analyses have provided key insights into the differences between these two mitochondrial populations, suggesting that mitochondria at synapses possess a proteomic profile predisposing them to greater vulnerability (Stauch et al., 2014). For example, a more significant accumulation of damage has been observed in the mitochondrial DNA of synaptic mitochondria, accompanied by changes in the regulation of critical factors such as mitochondrial transcription factor A (TFAM), nuclear respiratory factor 1 (NRF1), and PGC1-α. These changes affect essential processes such as mitochondrial DNA replication and maintenance, regulation of mitochondrial dynamics, and the ability to respond to calcium fluctuations at synapses (Stauch et al., 2014). Additionally, synaptic mitochondria exhibit alterations in the expression of proteins involved in mitochondrial fusion and fission, processes that are crucial for maintaining mitochondrial integrity and functionality. For example, a decrease in the expression of proteins such as mitofusin-1 (Mfn1) and Opa1, which are essential for fusion, has been reported, together with an increase in dynamin-1 (DNM1L), which regulates mitochondrial fission (Stauch et al., 2014). These imbalances could increase mitochondrial fragmentation, which could be necessary to enter synapses, but also could predispose to synaptic mitochondrial failure, decreasing bioenergetic efficiency and aggravating susceptibility to cumulative damage.

Our previous studies in animal models have revealed that, from 12 months of age, synaptic mitochondria show a marked decrease in ATP production, in contrast to non-synaptic mitochondria, which maintain their bioenergetic capacity for a longer time (Olesen et al., 2020; **Figure 3**). This initial decline could be associated with reduced expression of electron transport chain subunits essential for oxidative phosphorylation (Stauch et al., 2014). However, with advancing age, between 12 and 18 months, this decline in ATP production appears to slow down and eventually catch up with that of non-synaptic mitochondria (Olesen et al., 2020; **Figure 3**). This last could be because the expression of electron transport chain complex subunits decreases in non-synaptic mitochondria at 18 months. Similarly, the Ca^2+^ response capacity of synaptic mitochondria also declines at 12 months (Olesen et al., 2020; **Figure 3**). The vulnerability of synaptic mitochondria to altered Ca^2+^ transport is intrinsically linked to their role in regulating Ca^2+^ levels during neurotransmission. These organelles face high Ca^2+^ flux due to synaptic activity and must also handle these rapid increases efficiently to prevent cellular damage (Datta and Jaiswal, 2021). However, detailed proteomic studies have shown that these mitochondria exhibit decreased expression of proteins critical for Ca^2+^ transport, making them especially susceptible to Ca^2+^ overload and related stress (Stauch et al., 2014). In particular, MCU proteins and the calcium transport regulatory protein, MICU1, are significantly reduced in synaptic mitochondria compared to their non-synaptic counterparts (Stauch et al., 2014). MCU is a protein in the inner mitochondrial membrane that transports Ca^2+^ into the mitochondrial matrix, whereas MICU1 regulates the entry of Ca^2+^ through MCU (Gunter and Sheu, 2009). The lower expression of these proteins suggests a decreased ability to handle calcium in synaptic mitochondria, which may lead to excessive intracellular calcium accumulation and subsequent damage to mitochondrial and other cellular structures, even contributing to excitotoxicity.

A notable feature of synaptic mitochondria is their increased sensitivity to external insults, such as advanced glycation end products (Samanta et al., 2025). These compounds interfere with mitochondrial bioenergetics and increase the generation of ROS, contributing to oxidative stress and mitochondrial dysfunction (Patel et al., 2019; Chen et al., 2024). These alterations can disproportionately impact synaptic mitochondria due to their strategic location at synapses, where they must respond to rapid and continuous calcium fluctuations to maintain neurotransmission. Additionally, it has been described that Aβ (Quiroz-Baez et al., 2013) and phosphorylated tau (Torres et al., 2021a) accumulate specifically within synaptic mitochondria, exacerbating dysfunction (Du et al., 2010; Torres et al., 2022; Daniel Estrella et al., 2025). This phenomenon is particularly relevant in aging and neurodegenerative diseases, where synaptic mitochondria face a double challenge: deterioration induced by internal factors and cumulative damage by external agents.

To our knowledge, there is no evidence that synaptic mitochondrial dysfunction affects the levels of Aβ or tau in aging. However, some studies showed that mitochondria regulate cytosolic protein homeostasis (Ruan et al., 2017; Li et al., 2019), suggesting that it could contribute, almost in part, to the accumulation of abnormal proteins in aging and disease. For example, a study showed that inhibiting mitochondrial proteostasis increases the accumulation of α-synuclein and Aβ_42_ (Lautenschlager et al., 2020). Thus, mitochondrial dysfunction may impact Aβ and tau levels during aging; however, whether this is primarily or exclusively related to synaptic mitochondrial dysfunction is still unknown.

Preserving synaptic mitochondrial function is a promising target to prevent or attenuate age-related aging and neurodegenerative disease alterations. In fact, the relevance of synaptic mitochondria as a therapeutic target is supported by our previous studies demonstrating the benefits of mitochondria-targeted antioxidants, such as MitoQ and curcumin. These interventions have successfully mitigated the functional impairment of synaptic mitochondria without altering non-synaptic mitochondrial function, and this is sufficient to prevent cognitive deficits in aged murine models (Olesen et al., 2020). Although these findings suggest that MitoQ and curcumin could be potential therapeutic options for neurodegenerative diseases, their efficacy in such conditions has not yet been explored.

Furthermore, up to date, the study of synaptic mitochondria has been mainly focused on elucidating the differences with their non-synaptic counterpart and the importance of synaptic mitochondria on the development of age-related neurodegenerative diseases, which is the basis for future studies that could evaluate different therapeutic approaches targeting directly synaptic mitochondria. Until today, to our knowledge, there are studies using compounds to improve mitochondrial function, which have effects on both populations (Bertoni-Freddari et al., 1994; Olesen et al., 2020). However, studies targeting only the synaptic ones are not known.

Thus, synaptic mitochondria play an indispensable role in neuronal activity and face unique challenges due to their intrinsic vulnerability. Their bioenergetic fragility, sensitivity to external insults, and accumulation of genomic damage represent key factors in the progression of pathologies related to mitochondrial dysfunction. A detailed understanding of these functional and proteomic differences could offer new strategies to preserve mitochondrial health and prevent cognitive decline and neurodegenerative diseases.

## Conclusion and Future Directions

Synaptic mitochondria play a critical role in neuronal functioning, with their importance becoming more evident as the knowledge of aging and neurodegenerative diseases advances. Considering their specific localization at synapses and high functional bioenergetics and calcium homeostasis demands, synaptic mitochondria are especially susceptible to dysfunction. This vulnerability significantly contributes to aging-associated cognitive impairment and various neurological disorders. The changes in morphology and function that synaptic mitochondria undergo during aging highlight an urgent need for therapeutic strategies to preserve or re-establish their function. As we progress, it is crucial to decipher the mechanisms regulating mitochondria dynamics at synapses, such as mitochondrial transport, fusion, fission, and quality control. These mechanisms are critical for the mitochondrial function and integrity maintenance at synapses. Furthermore, a deep understanding of the differences between synaptic and non-synaptic mitochondria at molecular and functional levels could help develop specific therapies to beat the effects of aging and age-related diseases on neuronal function. Additional studies using different approaches are necessary to understand these differences. One of the best approaches is omics, specifically proteomic studies, to evaluate the overall changes in the expression of the different proteins, which could indicate changes in functionality. However, the precise differences in the functional outcome or the response to other stimuli could also be evaluated in the different isolated mitochondrial fractions as was done (Lores-Arnaiz and Bustamante, 2011; Lores-Arnaiz et al., 2016; Olesen et al., 2020). Finally, it could be evaluated the differences in the morphology of both mitochondrial populations using the same isolation protocols and transmission electron microscopy (Bertoni-Freddari et al., 1994; Torres et al., 2021). Such advancements could pave the way for treatments that slow down or even reverse the functional decline in neurons associated with synaptic mitochondrial dysfunction.

## Data Availability

*Not applicable*.
